# The Rehabilitation of Cognitive Impairment Following Stroke: A Commentary on a Systematic Review

**DOI:** 10.12968/bjnn.2023.19.Sup5.S6

**Published:** 2023-10-01

**Authors:** M Ghosh, O Hamer, JE Hill

**Affiliations:** Lancashire Teaching Hospitals NHS Foundation Trust; University of Central Lancashire

## Introduction

Stroke is regarded as one of the leading causes of death and disability worldwide (Katan et al. 2018). There are currently 100,000 individuals living with the after-effects of stroke within the United Kingdom (King et al. 2020). Post stroke cognitive impairment impacts on 20% to 80% of all stroke survivors (Sun et al. 2014). It is associated with increased dependence, poor rehabilitation outcomes, reduced quality of life and increased rates of institutionalisation (Lui et al, 2018). The rate of cognitive impairment can be influenced by a number of factors including age, education level, diagnostic criteria, geographical and race related factors (i.e., skin colour) (Sun et al, 2014). Despite the high prevalence and significant impact of cognitive impairment on post stroke functioning, there remains lack of clarity regarding interventions to support rehabilitation of cognitive impairment (O'Donoghue et al. 2022). Within the past decade, priority setting groups in the UK have deemed cognitive impairment as one of the top ten topics needing further research (Pollock et al. 2014). Furthermore, a recent study exploring risk factors, found atrial fibrillation to be the most significant risk factor for post-stroke cognitive impairment, pointing to the need for early screening and prevention of stroke (Obaid et al. 2020).

A key risk factor to health is the increased incidence of dementia following stroke (all types of stroke) (Ball et al, 2022). A recent systematic review found that severe stroke (National Institute of Health Stroke Scale>10) across all ages brought forward the incidence of dementia by 25 years, minor stroke by four years, and transient ischaemic attack by 2 years (Pendlebury and Rothwell. 2019). With a greater risk of dementia following stroke, routine cognitive screening, management and follow up for patients post stroke is needed to promote early diagnosis, prevent a loss of independence, and reduce mortality risk (Stolwyc et al. 2021; Gaynor et al. 2018).

Another common complication following stroke is that of delirium (Rollo et al. 2022). Delirium refers to as a transient period of impaired attention and consciousness which is seen in the acute post-stroke period (Rollo et al. 2022). The presence of delirium in the acute period post-stroke is responsible for cognitive and psychiatric disturbances up to three years after stroke (Nerdal et al. 2022). Delirium predicts significantly poorer global cognition and significantly high levels of neuropsychiatric symptoms which often cause challenging behaviour (such as anger, aggression, agitation, hallucination, delusion) (Nerdal et al. 2022). Delirium can also lead to further complications in that it is an independent risk factor for dementia 3 months post stroke (Dros et al. 2020). These studies highlight the negative impact delirium can have on the patient in the early and late stages of stroke, emphasising the need for prevention of delirium occurrences and proactive management post stroke.

Cognition is not a unitary concept, as it encompasses several processes across multiple domains (Sachdev et al, 2014). These include the ability to perceive, organise, assimilate, learn and generalise information which can impact on attention, memory, executive function, language and visuo- spatial ability (Abreu and Toglia. 1987; Toglia et al. 2009). These processes allow a person to identify and select information from their environment in order to function (Toglia et al. 2009). Cognitive ability underpins performance in a variety of daily living occupations and therefore it is important that patients undergo rehabilitation when cognition is affected by conditions such as stroke (Toglia 2011).

Cognitive rehabilitation involves functionally orientated remediation of these cognitive abilities (Cicerone et al. 2000). Cognitive rehabilitation is recommended by key organisations such as the National Institute for Care and Excellence, with guidelines focused on enhancing attention function, improving memory and self-awareness, as well as modifying the environment and activity to augment performance (NICE, 2013). Occupational therapy interventions aim to reduce activity limitations, enhancing participation in daily living and allowing individuals to live their life with an increased locus of control (Toglia et al. 2009; 2011). Although the goal is clear, there are several different approaches to rehabilitation (Obaid et al. 2020). Recent evidence suggests that a combination of cognitive and physical rehabilitation may have the most beneficial effects on post stroke cognitive impairment (Obaid et al. 2020). However, research has yet to establish or understand the longer-term impact of these interventions.

## Aim of commentary

This commentary aims to critically appraise the methods used within the review by O'Donoghue et al, (2022) and expand upon the findings in the context of clinical practice.

## Methods of the review by O'Donoghue et al

A total of seven databases were searched from inception to October 2019: PubMed, Embase, CENTRAL, PsycInfo, CiNAHL, the Vista and ClinicTrials.Gov. Only randomized & quasi-randomized control trials, feasibility studies and pilot studies were included. Studies were included if participants were adults aged >18 years with a clinical diagnosis of ischemic or haemorrhagic stroke, in the acute, subacute, or chronic stage poststroke (with or without a confirmed cognitive impairment). All studies which the primary or secondary aim was to improve cognitive function after stroke compared with standard care, no treatment control, waitlist control, or active control were included. Studies that were not published in English., were available in full texts and comprised of pharmacological interventions (including over-the-counter medications) were excluded.

Screening, data extraction and assessment of bias (Cochrane ‘Risk of Bias’ tool and The Grading of Recommendations Assessment, Development and Evaluation [GRADE]) were undertaken by two reviewers independently. Any disagreements were resolved by discussion among all authors until a consensus was reached. Where data was available, meta-analysis was employed to synthesise the data. Intervention effects were calculated using standardized mean differences (SMD) and 95% CI when different studies used different scales to assess the same outcome. Mean differences (MD) and 95% CI were employed when studies used the same scales to measure the same outcome.

## Results of the review by O'Donoghue et al

The systematic review included 64 studies involving 4005 participants. Within the studies, the mean age of participants was 62.5 years (ranging from 45 to 76 years). Among the sample of participants, the mean time post onset of stroke was 20.03 months (ranging from 48 hours to 6.25 years poststroke). Most studies were conducted in the acute phase (≤3 months poststroke; n= 20, 31%), and during the chronic stage (>6 months poststroke; n= 18, 28%). Studies recruited participants from inpatient acute setting, rehabilitation setting, community settings and from outpatient services. The studies within the review commonly included both ischemic and haemorrhagic types of stroke (n= 33). That said, several studies focused only on one type of stroke (n= 8). Many of the studies did not specify or report data relating to the type of stroke they included, with most just stating an inclusion criteria of ‘stroke’ patients (n= 23).

### Multiple Component Interventions

A meta-analysis revealed that the implementation of multiple component interventions resulted in improvement in memory scores (SMD, 0.49; 95% CI, 0.27–0.72; I^2^= 0%, four studies: RoB; three high, one moderate), functional status (SMD, 0.33 [95% CI, 0.05–0.62]; I^2^=61%, four studies: RoB; high risk of bias) general cognitive function at the end of treatment (MD, 1.56; 95% CI, 0.69–2.43; I^2^ =30%, three studies: RoB; two high, one moderate) and at <3 months poststroke (MD, 2.38 [95% CI, 0.97–3.80] I^2^=0%, three studies: RoB; two high, one moderate) when compared to standard care in adults post onset of stroke. There was no evidence of effect in attention, perception, depression, neglect, and quality of life.

### Cognitive Rehabilitation Interventions

The studies reported no evidence of any effect in the general cognitive function, memory, executive function, neglect, and quality of life comparing cognitive rehabilitation interventions (e.g., memory and executive function training incorporating memory strategies, education, goal setting and reading) with control group in adults post onset of stroke.

### Physical Activity Interventions

Meta analysis identified that physical activity interventions improved neglect scores compared with active control group of sham mirror therapy (<3 months poststroke; MD, 13.99 [95% CI, 12.67–15.32]; I^2^=0% two studies: RoB; two high risk) and improved balance scores compared with active control (stretching) (6 months poststroke; MD, 2.97 [95% CI, 0.71–5.23]; I^2^=0%, three studies: RoB; one high, two moderate) in adults post onset of stroke. There was no evidence of an effect in executive cognitive function comparing physical activity interventions with active control (stretching).

### Non-invasive brain stimulation interventions (NIBS)

A meta-analysis revealed that non-invasive brain stimulation interventions enhanced results of neglect using the line bisection test compared to a sham treatment in adults post onset of stroke (MD, 20.79 [95% CI, 14.53– 27.04]; I^2^=79%, three studies: RoB; one high, two moderate). A subgroup analysis of studies 3-6 months after stroke indicated that NIBS interventions improved neglect versus control (MD, 18.74 [95% CI, 11.50– 25.99]; I^2^=78% three studies: RoB; one high, two moderate).

There was also an increase in functional status compared to sham treatment (MD, 14.02 [95% CI, 8.41–19.62]; I^2^ =0% two studies: RoB; two moderate). However, when using the star cancellation test sham treatment improved measures of neglect (MD, −5.57 [95% CI, −8.53 to −2.61]; I^2^ =99% two studies: RoB; two moderate).

### Occupational-Based Interventions

There was no evidence of effect in general cognitive function or functional status comparing occupational-based interventions (workplace intervention programmes tailored according to the functional ability and workplace needs of the stroke survivor) with control group in adults post onset of stroke. Subgroup analysis revealed that studies <3 months poststroke showed an increase in general cognitive function from intervention group compared to control in adults post onset of stroke (MD, 0.39 [95% CI, 0.02– 0.76]; I^2^ =0%).

### Other Interventions

There was no evidence of effect in general cognitive function comparing prism adaptation therapy with control group in adults post onset of stroke (SMD, 0.40 [95% CI, −0.06 to 0.85]).

## Commentary

This systematic review scored 9 out of 11 on the Joanna Briggs (JBI) checklist (Aromataris et al. 2015). There were two criteria from the JBI checklist not discussed in the review which related to publication bias and recommendations to future research. It was evident that there was a lack of studies to fully assess the possibility of publication bias, however this influence was not discussed within the review. The paper details the different categories of rehabilitation interventions used for remediating post-stroke cognitive deficits and states that multi component interventions using physical and cognitive rehabilitation had good outcomes, for example, memory. However, it does not comment on the need for further research or suggest changes to policy, based on these findings. Despite these omissions, the study comprehensively categorises rehabilitation interventions, establishes effectiveness of these interventions, and acknowledges that whilst new information is being added to the evidence base, the study has its limitations. As such, it misses out on clearly articulating the areas of further research that are needed to improve this evidence base.

When identifying interventions for cognitive improvement it is important to firstly identify which outcomes are of importance to both patients and the rehabilitation process (Kyte et al. 2015). Multi-component interventions may improve memory scores, functional status, and general cognitive function after treatment, and up to three months poststroke. However, the review found no evidence of effect on attention, perception, depression, neglect, and quality of life. Similarly, physical activity-based interventions could improve neglect and balance scores, however there was no evidence of improvement for executive cognitive function. NIBS interventions demonstrate some potential regarding improving neglect and functional status but no evidence of improvement for general cognitive function.

The findings relating to the effectiveness of occupational therapy interventions of this systematic review are similar to a Cochrane review on Occupational Therapy interventions targeting cognitive impairments after stroke (Gibson et al. 2022). This review found that the effectiveness of occupational therapy interventions in cognitive impairment was unclear. Slight improvements were seen in global cognitive function, sustained attention, working memory and flexible thinking (Gibson et al. 2022). However, there is some uncertainty in regard to these estimations of effects due to high risk of bias and clinically important imprecision. The lack of high-quality evidence shows that recommendations for implementation of these interventions into clinical practise cannot yet be made.

It is important to note that no evidence of effect does not mean these interventions are not effective (Alderson 2004). From the findings of this review, it is evident that there have been relatively few studies conducted on these interventions, resulting in wide confidence intervals which makes it difficult to determine their effectiveness (or lack thereof). These wide confidence intervals indicate that we still have a considerable degree of uncertainty regarding the findings, even though at present, they suggest no evidence of effect. Specifically, there is still substantial uncertainty regarding the effects of multicomponent interventions on cognitive impairment, depression, and quality-of-life. Furthermore, there is uncertainty regarding the effect of cognitive rehabilitation interventions on memory, executive function, and neglect. There is also the need to consider the cognitive and behavioural sequelae of complications (such as delirium) and use the knowledge to design post-stroke delirium prevention programmes.

### CPD reflective questions

1. What are the key outcomes of importance to patients during the rehabilitation process which may guide the selection of intervention?
2. What are the practical considerations when establishing which intervention should be adopted for remediating cognitive deficits in adults post stroke?
3. Does the improvement of cognitive outcomes receive due importance and time, post stroke?
